# The expression of heterologous Fe (III) phytosiderophore transporter *HvYS1* in rice increases Fe uptake, translocation and seed loading and excludes heavy metals by selective Fe transport

**DOI:** 10.1111/pbi.12637

**Published:** 2016-10-10

**Authors:** Raviraj Banakar, Ána Alvarez Fernández, Javier Abadía, Teresa Capell, Paul Christou

**Affiliations:** ^1^ Departament de Producció Vegetal i Ciència ForestalUniversitat de Lleida‐Agrotecnio Center LleidaLleidaSpain; ^2^ Department of Plant NutritionAula Dei Experimental StationConsejo Superior de Investigaciones Científicas (CSIC)ZaragozaSpain; ^3^ ICREACatalan Institute for Research and Advanced StudiesBarcelonaSpain

**Keywords:** Rice, metal transporters, iron, toxic metals, barley YS1 transporter, 2′ deoxymugenic acid

## Abstract

Many metal transporters in plants are promiscuous, accommodating multiple divalent cations including some which are toxic to humans. Previous attempts to increase the iron (Fe) and zinc (Zn) content of rice endosperm by overexpressing different metal transporters have therefore led unintentionally to the accumulation of copper (Cu), manganese (Mn) and cadmium (Cd). Unlike other metal transporters, barley Yellow Stripe 1 (HvYS1) is specific for Fe. We investigated the mechanistic basis of this preference by constitutively expressing *HvYS1* in rice under the control of the *maize ubiquitin1* promoter and comparing the mobilization and loading of different metals. Plants expressing *HvYS1* showed modest increases in Fe uptake, root‐to‐shoot translocation, seed accumulation and endosperm loading, but without any change in the uptake and root‐to‐shoot translocation of Zn, Mn or Cu, confirming the selective transport of Fe. The concentrations of Zn and Mn in the endosperm did not differ significantly between the wild‐type and *HvYS1* lines, but the transgenic endosperm contained significantly lower concentrations of Cu. Furthermore, the transgenic lines showed a significantly reduced Cd uptake, root‐to‐shoot translocation and accumulation in the seeds. The underlying mechanism of metal uptake and translocation reflects the down‐regulation of promiscuous endogenous metal transporters revealing an internal feedback mechanism that limits seed loading with Fe. This promotes the preferential mobilization and loading of Fe, therefore displacing Cu and Cd in the seed.

## Introduction

Iron (Fe) is an important micronutrient for all living organisms (Winterbourn, 1995). Plants acquire Fe from the soil and mobilize it from the roots to the aerial organs to support essential processes such as photosynthesis, electron transport and respiration (Morrissey and Guerinot, 2009). Fe is also loaded into the seed endosperm to support germination (Lanquar *et al*., 2005) and thus becomes available as a micronutrient for humans. Rice is an important staple food crop, particularly in the developing world, but rice grains do not accumulate high levels of Fe, leading to severe Fe deficiency in populations that rely mostly on rice for their nutritional needs (Gómez‐Galera *et al*., 2010; Pérez‐Massot *et al*., 2013).

Metal acquisition and mobilization in plants are controlled by several families of membrane‐bound metal transporters (Hall and Williams, 2003; Vert *et al*., 2002) including the Fe‐regulated transporter (IRT), natural resistance‐associated macrophage protein (NRAMP), cation diffusion facilitator (CDF), yellow stripe‐like (YSL) and heavy metal ATPase (HMA) transporter families, as well as other Fe transporters in the chloroplast and vacuolar membranes (Duy *et al*., 2007; Hall and Williams, 2003; Vert *et al*., 2002; Zhang *et al*., 2012). Iron acquisition in rice involves different strategies for Fe^2+^ and Fe^3+^ (Ishimaru *et al*., 2006; Kobayashi and Nishizawa, 2012; Sperotto *et al*., 2012). In strategy I, Fe^2+^ ions are taken up into the root epidermis by OsIRT1/OsIRT2 in the plasma membrane (Ishimaru *et al*., 2006; Lee and An, 2009; Vert *et al*., 2002) and are then transported via the phloem and xylem to accumulate in the seeds (Ishimaru *et al*., 2010; Takahashi *et al*., 2011). Phloem transport involves the Fe^2+^ chelator nicotianamine (NA) and the YSL family transporters YSL2 and YSL16, whereas xylem transport involves NRAMP1 (Ishimaru *et al*., 2010; Takahashi *et al*., 2011) and the citrate efflux transporter FRD3 (Durrett *et al*., 2007). In strategy II, phytosiderophores (PS) such as mugineic acid (MA) and deoxymugenic acid (DMA) are secreted to the rhizosphere (Ma *et al*., 1999) where they solubilize Fe^3+^ by forming DMA‐Fe^3+^ complexes (Ma *et al*., 1999). The complex is taken up into the roots by YSL15 in the plasma membrane (Inoue *et al*., 2009). The DMA‐Fe^3+^ complex is transported through the phloem by YSL18 and accumulates in the seeds in the same form (Ayoma *et al*., 2009), whereas translocation through the xylem is also mediated by the citrate efflux transporter FRDL1 (Yokosho *et al*., 2009). Rice, which is adapted for growing in anaerobic soils where Fe is more soluble, produces much less PS than barley, which is adapted to alkaline soils. In fact, rice is the only cereal species that combines components of strategy I plants (OsIRT1 and OsIRT2; Ishimaru *et al*., 2006) with PS production and Fe‐PS uptake (OsYSL15; Inoue *et al*., 2009).

Although Fe is abundant in the soil, rice has only a limited ability to acquire and mobilize Fe and load it into the endosperm (Lee and An, 2009; Lee *et al*., 2009) most likely due to the weak expression of Fe transporters in the root (Inoue *et al*., 2009; Lee and An, 2009; Lee *et al*., 2009; Tan *et al*., 2015). Previous efforts to increase the uptake of Fe into rice plants have therefore focused on the overexpression of metal transporters (Bashir *et al*., 2013). However, most transporters are promiscuous and those responsible for the mobilization of Fe may also transport Zn (another important micronutrient) and other metals such as Cu, Mn, Ni and Cd, some of which are toxic even at low levels (Hall and Williams, 2003; Ishimaru *et al*., 2010; Takahashi *et al*., 2011; Thomine and Vert, 2013; Vert *et al*., 2002). The overexpression of *OsIRT1, OsIRT2, MxIRT1, AtIRT1, OsYSL15* and *OsYSL2* in rice therefore increased the levels of Zn, Cu, Mn, Cd and Ni mobilized from the soil and this was shown to be detrimental to plant health (Lee and An, 2009; Nishida *et al*., 2011; Tan *et al*., 2015; Uraguchi and Fujiwara, 2012).

One approach that can address this challenge is the overexpression of heterologous metal transporters that are selective for Fe, with no affinity for other divalent cations (Clemens *et al*., 2013; Slamet‐Loedin *et al*., 2015). The barley (*Hordeum vulgare*) YS1 protein (HvYS1) is an Fe‐selective metal transporter expressed in the root epidermal cells (Murata *et al*., 2006, 2008, 2015). HvYS1 expression is induced by Fe deficiency but not by the depletion of other metals (Ueno *et al*., 2009). Yeast complementation studies have shown that HvYS1 is a strict DMA‐Fe^3+^ transporter that does not interact with Zn, Cu, Mn or Cd complexed with DMA or metals complexed with NA (Murata *et al*., 2006). Hence, this selectivity is attributed to an Fe‐specific outer membrane loop between the sixth and seventh transmembrane domains (Murata *et al*., 2008).

Here, we investigated the mechanism by which HvYS1 promotes the selective transport of Fe using rice as a model. The heterologous expression of HvYS1 improved Fe uptake and root‐to‐shoot translocation, and achieved a moderate increase in Fe seed loading, without increasing the uptake and root‐to‐shoot translocation of Zn, Cu or Mn. The concentrations of Zn and Mn in the seed were unaffected by HvYS1 expression, whereas the concentration of Cu declined. Cadmium uptake, root‐to‐shoot translocation and seed loading were also inhibited in these plants. The preferential mobilization of Fe at the expense of other metals reflects the inhibition of heavy metal seed loading due to the selective transport of Fe by HvYS1.

## Results

### The constitutive overexpression of HvYS1 in rice improves Fe uptake, translocation and seed loading

**Figure 1:**
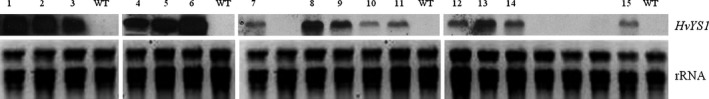
RNA blot analysis showing transgene expression in the leaf tissue of wild‐type (WT) and transgenic lines expressing *HvYS1*. rRNA: ribosomal RNA;* HvYS1*: barley yellow stripe 1 transporter.

We co‐transformed 7‐day‐old mature seed‐derived zygotic rice embryos with a plasmid containing *HvYS1* driven by the constitutive maize ubiquitin 1 (*ubi‐1*) promoter and another plasmid carrying the selectable marker *hpt* driven by the *CaMV35S* promoter and regenerated transgenic plants under hygromycin selection. *HvYS1* expression in 15 independent transgenic lines was confirmed by RNA blot analysis (Figure 1). These lines and corresponding wild‐type plants were grown to maturity and T_1_ seeds were collected. The five transgenic lines with the highest levels of *HvYS1* expression were bred to homozygosity for detailed analysis.

**Figure 2:**
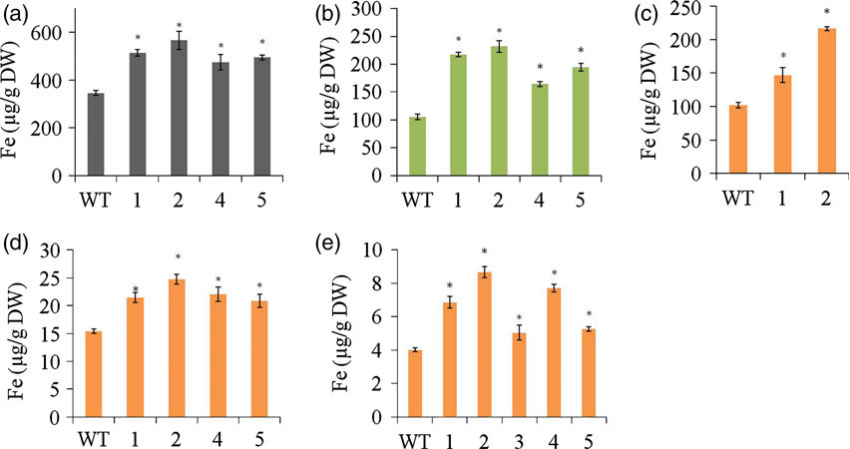
Concentrations of Fe (μg/g DW) in roots (a), leaves (b), husks (c), unpolished seeds (d) and polished seeds (e) of wild‐type (WT) and T_2_ generation transgenic lines expressing *HvYS1* (lines 1, 2, 3, 4, 5). Asterisks indicate a statistically significant difference between wild‐type and transgenic plants as determined by Student's *t*‐test (*P *<* *0.05; *n* = 6). DW: dry weight. Iron measurements in husk were taken from two representative transgenic lines.

We hypothesized that constitutive *HvYS1* expression might improve Fe uptake, root‐to‐shoot translocation and seed loading in the transgenic lines because HvYS1 is a specific Fe transporter in barley expressed in root epidermal cells and achieves Fe (III)‐PS translocation when expressed in yeast (Murata *et al*., 2006), *X. laevis* oocytes (Murata *et al*., 2008) and petunia (Murata *et al*., 2015). Accordingly, the T_2_ *HvYS1* transgenic lines contained up to 1.6‐fold more Fe in the roots than wild‐type controls, that is 566 ± 38 vs 345 ± 10 μg Fe/g dry weight (DW) (Figure 2a). This in turn enhanced the root‐to‐shoot translocation of Fe in the transgenic lines, resulting in up to 2.2‐fold more Fe in the leaves, that is 231 ± 10 vs 104 ± 5 μg Fe/g DW (Figure 2b). This increase in Fe uptake and root‐to‐shoot translocation also had an impact on Fe seed loading. The husks of the transgenic seeds contained up to 2.1‐fold more Fe than wild‐type seeds: 216 ± 3 vs 102 ± 4 μg Fe/g DW (Figure 2c). The unpolished transgenic seeds contained up to 1.6‐fold more Fe than wild‐type seeds: 24.0 ± 0.5 vs 15.4 ± 0.4 μg Fe/g DW (Figure 2d), whereas the polished transgenic seeds (the endosperm) contained 2.1‐fold more Fe than wild‐type endosperm: 8.7 ± 0.3 vs 4.0 ± 0.1 μg/g DW Fe (Figure 2e). These results suggest that *HvYS1* expression in the transgenic lines improved Fe mobilization from the soil to the roots, root‐to‐shoot translocation and seed loading, with loading of Fe occurred preferentially into the endosperm rather than into the bran.

### DMA synthesis and accumulation are enhanced in the HvYS1 transgenic plants

**Figure 3:**
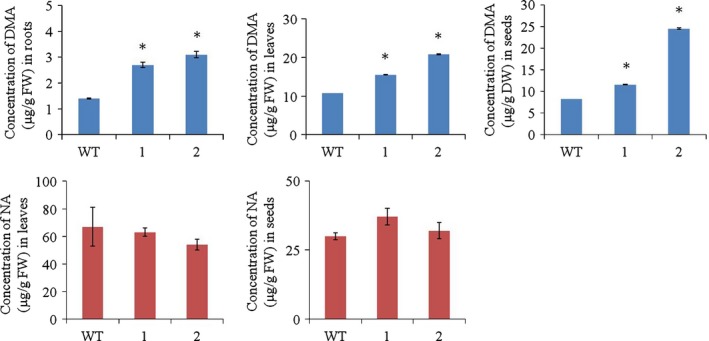
Concentration of 2′‐deoxymugenic acid (DMA) and nicotianamine (NA) (μg/g FW) in roots, leaves and polished seeds of wild‐type (WT) and two selected T_2_ generation transgenic lines expressing *HvYS1* (lines 1, 2). Asterisks indicate a statistically significant difference between wild‐type and transgenic plants as determined by Student's *t*‐test (*P *<* *0.05; *n* = 3). NA levels in the roots were below the detection limit. FW: fresh weight.

Rice produces DMA (Araki *et al*., 2015), and HvYS1 transports Fe^3+^ as a complex with DMA and MA with the same efficiency (Murata *et al*., 2008). We therefore hypothesized that the higher levels of Fe in the transgenic lines should be accompanied by higher levels of DMA. We measured the amount of DMA in the roots, leaves and seeds of selected T_2_ *HvYS1* transgenic lines and observed significantly higher levels of DMA in all three tissues compared to wild‐type plants (Figure 3a, b, c). These data confirm that the increased mobilization of Fe in the transgenic plants coincides with higher levels of DMA, indicating that the additional Fe is likely to be mobilized as an Fe^3+^‐DMA complex. We then measured the levels of NA in the tissues where we measured DMA to investigate whether the expression of *HvYS1* followed by Fe^3+^‐DMA transport influences NA levels. Although the quantification of NA was not possible in roots as the levels were below the detection limit, transgenic lines did not differ significantly from wild type for NA levels in leaves and seeds (Figure 3d, e, f). The data indicate that endogenous NA synthesis and accumulation were not influenced due to Fe^3+^‐DMA transport by *HvYS1*.

### The selective mobilization of Fe by *HvYS1* does not affect the uptake or root‐to‐shoot translocation of Zn, Cu and Mn

**Figure 4:**
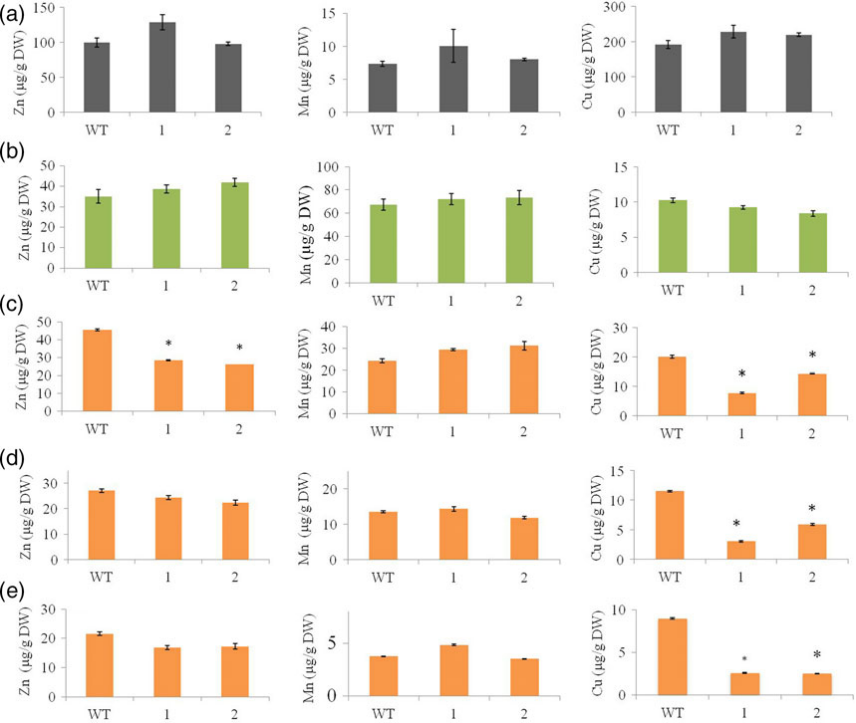
Concentrations of Zn (left), Mn (middle) and Cu (right), all in μg metal per g DW, in roots (a), leaves (b), husks (c), unpolished seeds (d) and polished seeds (e) of wild‐type (WT) and two selected T_2_ generation transgenic lines expressing *HvYS1* (lines 1, 2). Asterisks indicate a statistically significant difference between wild‐type and transgenic plants as determined by Student's *t*‐test (*P *<* *0.05; *n* = 6). DW: dry weight.

As many Fe transporters can also transport Zn, Cu and Mn (Lee *et al*., 2009), we investigated the distribution of these three metals in the *HvYS1* transgenic lines to confirm the specificity of the transporter in its heterologous environment. We found no difference in the distribution of these three metals when comparing transgenic and wild‐type roots (Figure 4a) and leaves (Figure 4b) suggesting that *HvYS1* achieves the selective uptake and root‐to‐shoot translocation of Fe and excludes Zn, Cu and Mn.

### The selective mobilization of Fe by *HvYS1* does not affect seed loading with Mn but influences the distribution of Zn in the husk and Cu in the endosperm

In contrast to the straightforward metal distribution profile in the vegetative tissues, the impact of *HvYS1* on metal distribution in the seeds was more complex. We compared the distribution of metals in the husk (Figure 4c), unpolished (Figure 4d) and polished seeds (Figure 4e). We found no difference between the transgenic and wild‐type seeds in terms of Mn loading, suggesting the distribution of Mn among the different seed tissues was unaffected by the moderate increase in Fe loading caused by the expression of *HvYS1*. However, Zn was specifically displaced from the husk in the transgenic lines, resulting in a 1.7‐fold depletion, from 45 ± 1 down to 26 ± 2 μg Zn/g DW (Figure 4c), although there was no significant difference in Zn levels when we compared the unpolished or polished transgenic and wild‐type seed. In contrast to the situation for Mn and Zn, we found that Cu was depleted in all three seed tissues in the transgenic lines. The transgenic husk contained 7.8 ± 0.3 μg Cu/g DW compared to 20 ± 0.6 μg Cu/g DW in the wild‐type husk, reflecting a 2.5‐fold decrease in Cu (Figure 4c). The unpolished transgenic seed contained 3.03 ± 0.1 μg Cu/g DW, 3.7‐fold lower than the wild‐type level of 11.5 ± 0.1 μg Cu/g DW (Figure 4d). Finally, the polished transgenic seed contained 2.5 ± 0.1 μg/g DW Cu, 3.8‐fold lower than the wild‐type level of 9 ± 0.1 μg Cu/g DW (Figure 4e). The lower levels of Zn and Cu in the transgenic seeds suggest that the increase in the delivery of Fe selectively suppresses Zn accumulation in the husk and Cu accumulation in all seed tissues, with the effect being particularly intense in the endosperm.

### The selective mobilization of Fe in the transgenic lines suppresses the mobilization of Cd at all steps along the translocation pathway

**Figure 5:**
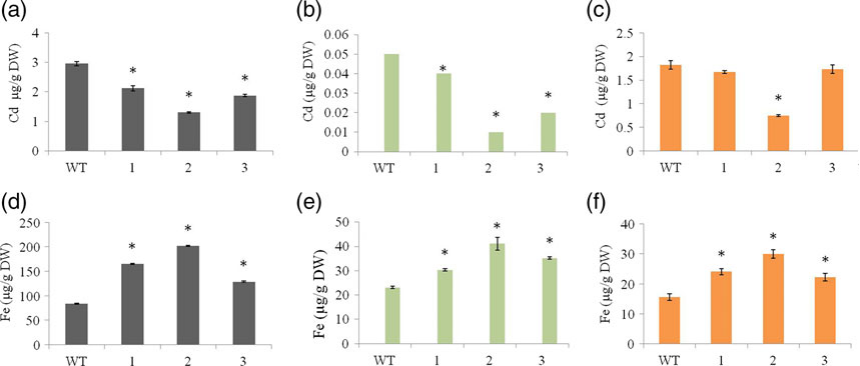
Concentrations of Cd (top row) and Fe (bottom row), both in μg/g DW, in (a and d) roots, (b and e) leaves and (c and f) unpolished seeds of wild‐type (WT) and T_3_ generation transgenic lines expressing *HvYS1* (lines 1, 2, 3) supplied with 10 μm CdCl_2_. Asterisks indicate a statistically significant difference between wild‐type and transgenic plants as determined by Student's *t*‐test (*P *<* *0.05; *n* = 6). DW: dry weight.

Many Fe transporters not only transport other divalent cations such as Zn, Mn and Cu, but also toxic metals such as Cd (Lee *et al*., 2009; Takahashi *et al*., 2011). We therefore compared the distribution of Fe and Cd in the transgenic lines and wild‐type controls when Cd was added to the soil to gain more insight into the selective mobilization of different metals by HvYS1 in its heterologous environment. Unlike Zn, Mn and Cu, whose distribution in vegetative tissues was unaffected, we found that the transgenic lines contained significantly lower levels of Cd than wild‐type plants in the roots and leaves as well as the seeds (Figure 5). When compared to the wild type, the transgenic lines accumulated 2.3‐fold less Cd in roots (Figure 5a), fivefold less Cd in leaves (Figure 5b) and 2.4‐fold less Cd in unpolished seeds (Figure 5c). In contrast, when compared to the wild type, the transgenic lines contained 2.4‐fold more Fe in roots (Figure 5d), 1.8‐fold more Fe in leaves (Figure 5e) and 1.9‐fold more Fe in seeds (Figure 5f). These data suggest that Fe mobilized by HvYS1 in the roots and shoots suppresses the uptake and translocation of Cd and that Fe delivery to the seeds also prevents seed loading with Cd and/or displaces Cd that is already in situ.

### Homeostasis mechanisms limit Fe seed loading in the transgenic lines

The selective mobilization of Fe by HvYS1 in the transgenic lines leads to a moderate increase in Fe levels in the seeds. A possible explanation for the modest increase in Fe levels in transgenic lines is that an Fe homeostasis mechanism imposes limitations on Fe accumulation. Fe homeostasis in rice involves a number of genes controlling uptake, root‐to‐shoot translocation, remobilization from the flag leaf and deposition in seeds (Table S1) suggesting that these endogenous genes may be modulated by the heterologous expression of *HvYS1*. To investigate the influence of HvYS1 on the expression of endogenous Fe homeostasis genes, we measured the expression of genes controlling Fe uptake (*OsIRT1*,* OsYSL15* and *OsNRAMP5*), long‐distance transport (*OsFRDL1*,* OsYSL2*,* OsYSL16*,* OsYSL18* and *OsNRAMP1*), vacuolar sequestration (*OsVIT1*), storage (*OsFERRITIN1*), endogenous phytosiderophore synthesis pathway (*OsSAMS1, OsNAS2, OsNAS3, OsNAAT1, OsDMAS1*) and transcription factor (*OsIDEF1*) in the roots, leaves and seeds of the *HvYS1* transgenic lines and wild‐type controls (Table S1).

In the roots, *OsIRT1* and *OsYSL15* (controlling Fe uptake) were down‐regulated by 3‐fold and 2.2‐fold, respectively, in the transgenic lines (Table 1; Figure S1). Furthermore, the Fe^2+^‐NA transporter *OsYSL16* was down‐regulated by 3.7‐fold, the Fe^3+^‐citrate transporter *OsFRDL1* was down‐regulated by 5.1‐fold, the vacuolar transporter *OsVIT1* was down‐regulated by 7.7‐fold, iron storage *OsFERRITIN1* was down‐regulated by 5.6‐fold, and the transcription factor regulating metal homeostasis *OsIDEF1* was down‐regulated by 3‐fold (Table 1; Figure S1). Our results suggest that endogenous Fe uptake and root‐to‐shoot translocation are down‐regulated by *HvYS1* expression. The expression of *OsNRAMP5* and *OsNRAMP1* was up‐regulated by 3.2‐fold and 4.4‐fold, respectively, in the transgenic lines compared to wild‐type controls (Table 1; Figure S1). This suggests that these genes were up‐regulated to balance Fe uptake and translocation. Among the endogenous PS synthesis genes, expression of *OsSAMS1* (6.4‐fold), *OsDMAS1* (2‐fold) was up‐regulated, whereas the expression of *OsNAS2* (17‐fold)*, OsNAS3* (1.7‐fold) *and OsNAAT1* (2.5‐fold) was down‐regulated. This suggests that the expression of *HvYS1* modulates expression of genes for the conversion of L‐methionine to S‐adenosyl methionine (*OsSAMS1*) and 3′‐keto intermediate to DMA (*OsDMAS1*) but such an alteration in endogenous PS pathway suppresses the expression of genes for the conversion of S‐adenosyl methionine to NA (*OsNAS2, OsNAS3*) and NA to 3′‐keto intermediate (*OsNAAT1*).

**Table 1:** Fold change in the relative expression level of *OsIRT1, OsYSL15, OsNRAMP5, OsVIT1, OsYSL2, OsYSL16, OsFRDL1, OsYSL18, OsNRAMP1, OsFERRITIN1, OsSAMS1, OsNAS2, OsNAS3, OsNAAT1, OsDMAS1 and OsIDEF1* in roots (left), flag leaf (centre) and seeds (right) at grain filling stage in wild‐type (WT) and T_2_ generation transgenic lines expressing *HvYS1* (Line 1 and Line 2). Arrows show up‐regulation and down‐regulation. Gene‐specific primers are listed in Table S2. NC, no change; ND, not determined

Genes		Roots	Flag leaf	Seeds
Metal uptake	*OsIRT1*	↓3	*1.8* **↑**	*2.8*↓
	*OsYSL15*	↓2.2	*ND*	*7.2*↓↓
	*OsNRAMP5*	**↑**3.2	*2.6* **↑**	*NC*
Vacuolar sequestration	*OsVIT1*	↓↓7.7	*NC*	*9*↓↓
Long‐distance transport	*OsYSL2*	NC	*NC*	*3.4*↓
	*OsYSL16*	↓3.7	*3* **↑**	*NC*
	*OsFRDL1*	↓↓5.1	*NC*	*2.7*↓
	*OsYSL18*	NC	*2.2* **↑**	*7.5*↓↓
	*OsNRAMP1*	**↑**4.4	*NC*	*NC*
Iron storage	*OsFERRITIN1*	↓5.6	*3.1* **↑**	*NC*
Endogenous phytosiderophore synthesis pathway	*OsSAMS1*	6.4**↑**	*2.2*↓	*NC*
	*OsNAS2*	17↓↓↓↓	*3.2* **↑**	*1.8* **↑**
	*OsNAS3*	1.7↓	*9* **↑↑**	*3.4* **↑**
	*OsNAAT1*	2.5↓	*2.2* **↑**	*2.1*↓
	*OsDMAS1*	2**↑**	*2.2* **↑**	*2.5*↓
Transcription factor	*OsIDEF1*	3↓	*1.8* **↑**	*2.5*↓

In the leaves, *OsIRT1*,* OsNRAMP5*,* OsYSL16*,* OsYSL18*,* OsFERRITIN1* and *OsIDEF1* were all up‐regulated in the transgenic lines by between 1.8‐fold and 3.1‐fold (Table 1; Figure S1), indicating that Fe remobilization from leaves and storage was enhanced in the transgenic lines. Similarly, expression of *OsNAS2, OsNAS3, OsNAAT1 and OsDMAS1* was up‐regulated by 3.2‐, 9‐, 2.2‐ and 2.2‐fold, respectively, in the transgenic lines compared to the wild type (Table 1; Figure S1). In contrast, expression of *OsSAMS1* was down‐regulated by 2.2‐fold in the transgenic lines compared to wild type (Table 1; Figure S1). These results suggest that generally the expression of endogenous PS pathway genes was modulated to enhance Fe mobilization. In the seeds, *OsIRT1, OsYSL15, OsYSL2, OsYSL18, OsFRDL1*,* OsVIT1* and *OsIDEF1* were down‐regulated by 2.8‐fold, 7.2‐fold, 3.4‐fold, 7.5‐fold, 2.7‐fold, ninefold and 2.5‐fold, respectively (Table 1; Figure S1), suggesting that endogenous genes that promote Fe accumulation are down‐regulated to limit Fe accumulation in the seeds. In contrast to the general down‐regulation of metal transporters, the expression of *OsNAS2* and *OsNAS3* was up‐regulated by 1.8‐ and 3.4‐fold, respectively, whereas *OsNAAT1* and *OsDMAS1* expressions were down‐regulated by 2.1‐ and 2.5‐fold, respectively (Table 1; Figure S1). These results suggest that the conversion of NA to the 3′‐keto intermediate (by *OsNAAT1*) followed by the latter's conversion to DMA (by *OsDMAS1*) was down‐regulated to limit Fe accumulation in seeds.

## Discussion

Rice plants secrete DMA from the root surface (Suzuki *et al*., 2008), which chelates Fe^3+^ in the soil allowing the resulting Fe^3+^‐DMA complex to be taken up by Fe^3+^‐DMA transporters. The complexes are translocated internally and ultimately accumulate in the seeds (Inoue *et al*., 2009). One strategy to enhance Fe uptake, translocation and accumulation is therefore to overexpress appropriate metal transporters. However, by and large metal transporters are promiscuous and they can transport toxic metals such as Cd, along with metals that are essential nutrients. The broad impact of heterologous metal transporter overexpression on metal accumulation in seeds, and the expression of endogenous genes involved in metal homeostasis, is thus still unclear because the mechanisms of metal homeostasis in plants are complex and they depend on many different factors.

To address these issues in more detail, we generated transgenic rice plants overexpressing the barley Fe^3+^‐DMA transporter HvYS1, which is strictly specific for Fe and therefore allows studying the impact on Fe levels. The constitutive expression of *HvYS1* increased Fe uptake from the soil, root‐to‐shoot translocation and seed loading, resulting in concentration increases of 1.6‐, 2.2‐ and 2.1‐fold, respectively, in the roots, leaves and endosperm of the T_2_ transgenic plants. The transgenic lines also accumulated significantly higher levels of DMA in the roots, leaves and seeds. Similar results were reported by others when the Fe^2+^ transporter genes *OsIRT1*,* MxIRT1* and *AtIRT1* were expressed in rice, as well as the Fe^3+^‐DMA transporter gene *OsYSL15* and the promiscuous metal transporter gene *OsNRAMP5* (whose product can transfer Fe, Mn and Cd), but the increase in endosperm Fe levels was more moderate, leading to concentration increases of 1.2‐ to 1.3‐fold when compared to wild‐type seeds (Boonyaves *et al*., 2016; Ishimaru *et al.,* 2012; Lee and An, 2009; Lee *et al*., 2009; Tan *et al*., 2015). This suggests that the overexpression of *HvYS1* enhances Fe^3+^‐DMA uptake, root‐to‐shoot translocation and seed loading more efficiently than the other genes, resulting in a 2.1‐fold increase in Fe levels in the endosperm (i.e. from 4 μg Fe/g DW in wild‐type plants to 8.7 μg Fe/g DW in the transgenic lines). Compared to other cereals, barley is highly tolerant to Fe deficiency and the presence of the efficient Fe transporter YS1 in the plasma membrane may explain this phenomenon (Murata *et al*., 2006, 2008).

Next, we investigated the impact of heterologous *HvYS1* expression on Zn, Mn and Cu uptake, root‐to‐shoot translocation and seed accumulation. The *HvYS1* lines did not differ significantly from wild‐type plants in terms of the concentration of Zn, Mn and Cu in the roots and leaves. Similarly, *HvYS1* expression in *Xenopus laevis* oocytes revealed that HvYS1 has the ability to transport Fe^3+^‐MA complexes but not complexes with other metals (Murata *et al*., 2006, 2008). In contrast, genes encoding the promiscuous metal transporters OsIRT1, MxIRT1, OsNRAMP5, OsHMA3 and AtIRT1 increased the levels of Zn, Mn and Cu, respectively, by 1.3‐, 1.2‐ and 1.4‐fold in rice roots, and by 1.4‐, 1.2‐ and 1.3‐fold in rice leaves (Boonyaves *et al*., 2016; Ishimaru *et al.,* 2012; Lee and An, 2009; Lee *et al*., 2009; Tan *et al*., 2015; Ueno *et al*., 2010). There was no difference in the distribution of Zn and Mn in the unpolished and polished seeds of the transgenic lines compared to wild‐type seeds, but the concentration of Cu was 3.8‐fold lower in the transgenic seeds. The overexpression of *OsIRT1*,* MxIRT1*,* OsHMA3*,* OsNRAMP5* and *AtIRT1* increased the concentrations of Zn, Mn and Cu in rice seeds by 1.5‐, 1.3‐ and 1.6‐fold, respectively (Boonyaves *et al*., 2016; Ishimaru *et al.,* 2012; Lee and An, 2009; Lee *et al*., 2009; Tan *et al*., 2015; Ueno *et al*., 2010). The promiscuous transporters increase seed loading with Zn, Mn and Cu by directly transporting these metals into the seed, whereas the specificity of HvYS1 means that only Fe is loaded and any differences in other metals must be attributed to passive effects, that is Cu being passively displaced by Fe in the *HvYS1* transgenic rice plants. Zinc is nutritionally important for human health, whereas Mn and Cu are toxic even at moderate levels (Alimba *et al*., 2016). The selective loading of Fe into the endosperm of the *HvYS1* lines is therefore advantageous over the general increase in metal levels previously achieved by the overexpression of promiscuous transporters (Boonyaves *et al*., 2016; Ishimaru *et al.,* 2012; Lee and An, 2009; Lee *et al*., 2009; Tan *et al*., 2015; Ueno *et al*., 2010).

The expression of *HvYS1* doubled the concentration of Fe in the transgenic seeds compared to wild‐type seeds, an effect similar to those achieved by expressing *OsIRT1, AtIRT1, MxIRT1, OsYSL15* or *OsNRAMP5* (Boonyaves *et al*., 2016; Ishimaru *et al.,* 2012; Lee and An, 2009; Lee *et al*., 2009; Slamet‐Loedin *et al*., 2015; Tan *et al*., 2015; Ueno *et al*., 2010). This suggests there may be a limit to the amount of Fe that can be deposited in the seed, because metal homeostasis mechanisms are tightly regulated and do not allow Fe accumulation beyond certain limits (Sperotto *et al*., 2012; Wang *et al*., 2013). Hence, we investigated the impact of *HvYS1* on the expression of endogenous genes controlling Fe mobilization, including the Fe‐regulated metal uptake transporters encoded by *OsIRT1* (Fe‐Zn‐Mn), *OsYSL15* (Fe^3+^‐DMA) and *OsNRAMP5* (Fe‐Mn); the vacuolar Fe‐Zn transporter encoded by *OsVIT1*; long‐distance transporters encoded by *OsYSL2* (Fe‐Mn), *OsYSL16* (Fe), *OsNRAMP1* (Fe), *OsFRDL1* (Fe^3+^‐citrate) and *OsYSL18* (Fe^3+^‐DMA), Fe storage protein ferritin encoded by *OsFERRITIN1*, genes involved in PS synthesis such as *OsSAMS1, OsNAS2, OsNAS3, OsNAAT1, OsDMAS1* and finally *OsIDEF1* a transcription factor regulating Fe homeostasis. This allowed us to unravel facets of the mechanism through which Fe accumulation in the seeds is regulated and how the homeostasis mechanism operating in different tissues regulates Fe accumulation in roots, leaves and seeds.

In the roots of the *HvYS1* transgenic lines, *OsIRT1* and *OsYSL15* were slightly down‐regulated. These encode Fe‐regulated transporters and the corresponding genes are induced by Fe deficiency and repressed when Fe levels are sufficient (Inoue *et al*., 2009; Lee and An, 2009). Therefore, the higher Fe levels in the transgenic lines appear to create an Fe‐sufficient environment causing these two genes to be suppressed. OsIRT1 carries Zn and Mn in addition to Fe, so the down‐regulation of *OsIRT1* may trigger the expression of the Fe‐Mn transporter *OsNRAMP5* to increase the uptake of Mn. Iron mobilization from the roots through the xylem promotes Fe seed loading (Yoneyama *et al*., 2015). The transporter OsNRAMP1 loads the xylem with Fe (Takahashi *et al*., 2011), and OsNRAMP5 promotes both Fe uptake and xylem loading (Yang *et al*., 2014). The up‐regulation of these two transporters in the *HvYS1* lines therefore suggests an increase in Fe xylem loading and root‐to‐shoot translocation. DMA plays a major role in uptake and root‐to‐shoot translocation of Fe in rice (Bashir *et al*., 2014). Synthesis of S‐adenosyl methionine (SAM) from L‐methionine is carried out by *OsSAMS1*, and NA is synthesized from SAM through expression of *OsNAS2 and OsNAS3*. NA is then converted to a 3′‐keto intermediate by *OsNAAT1*, and finally, *OsDMAS1* catalyses the formation of DMA through the 3′‐keto intermediate precursor molecule (Bashir *et al*., 2014). In *HvYS1* lines, expression of *OsSAMS1* and *OsDMAS1* was up‐regulated, whereas expression of *OsNAS2, OsNAS3 and OsNAAT1* was down‐regulated. Expression of *OsSAMS1, OsNAS2, OsNAS3, OsNAAT1 and OsDMAS1* was up‐regulated in roots under Fe deficiency, while the reverse was true under Fe sufficiency conditions (Bashir and Nishizawa, 2006; Bashir *et al*., 2014; Inoue *et al*., 2003, 2008). Therefore, in *HvYS1* lines, up‐regulation of *OsDMAS1* increased DMA levels due to increased Fe levels in roots. The down‐regulation of *OsNAS2, OsNAS3* and *OsNAAT1* indicates that the Fe homeostasis mechanism operates to restrict Fe uptake and root‐to‐shoot translocation by limiting the synthesis of NA and its conversion into DMA.

The remobilization of Fe from the flag leaf through the phloem is important for seed loading (Curie *et al.,* 2009; Yoneyama *et al*., 2015), and this is facilitated by the transporters encoded by *OsYSL16* (Kakei *et al*., 2012) and *OsYSL18* (Ayoma *et al*., 2009). *OsYSL16* and *OsYSL18* were up‐regulated in the transgenic lines, suggesting an increase in phloem loading with Fe, resulting in higher Fe levels in the seeds. Similar to its role in uptake and root‐to‐shoot translocation of Fe, DMA is also important in the remobilization of Fe from flag leaf to seeds (Ayoma *et al*., 2009; Masuda *et al*., 2009). *OsNAS2, OsNAS3, OsNAAT1* and *OsDMAS1* were up‐regulated in *HvYS1 lines*. The up‐regulation of *OsNAS2, OsNAS3, OsNAAT1* and *OsDMAS1* suggests increased synthesis and accumulation of DMA in flag leaf leading to enhanced Fe remobilization from flag leaf in transgenic lines compared to wild type. The Fe storage protein ferritin is also regulated by the amount of Fe present in the cell (Jain and Connolly, 2013). The induction of *OsFERRITIN1* in the flag leaf suggests that Fe in the flag leaf was not freely available for remobilization through the phloem because Fe is diverted to the chloroplast (Long *et al*., 2008). Iron storage as a complex with ferritin therefore appears to act as a buffer to control the remobilization of Fe through the phloem (Long *et al*., 2008). *OsIRT1, OsYSL15, OsFRDL1* and *OsYSL18* were down‐regulated in the transgenic seeds, which was surprising because all four corresponding proteins are known to contribute to Fe seed loading. Indeed, the suppression of *OsYSL15* and *OsFRDL1* expressions resulted in 1.5‐fold and 1.3‐fold lower levels of Fe in rice seeds, respectively (Lee *et al*., 2009; Yokosho *et al*., 2009), whereas the overexpression of *OsIRT1* increased Fe levels in the seed by 1.3‐fold, with OsYSL18 proposed to facilitate Fe loading into the phloem (Ayoma *et al*., 2009; Lee and An, 2009). Similar to the metal transporters, expression of *OsNAAT1* and *OsDMAS1* was down‐regulated in the transgenic lines. DMA is important for Fe seed loading (Masuda *et al*., 2009). Therefore, limited loading of Fe in the transgenic seeds suggests that homeostasis is triggered once a certain threshold is reached, which involves the down‐regulation of genes encoding endogenous transporters and DMA synthesis responsible for the mobilization of Fe. This mechanism operates in the roots, flag leaf and seeds. Similarly, rice engineered to produce higher levels of phytosiderophores increased only fourfold the wild‐type level of Fe in the seeds, due to the modulation of genes controlling metal uptake, translocation and seed loading (Wang *et al*., 2013; Banakar *et al*. under review).

Increasing the loading of seeds with Fe decreased the seed concentrations of Cd. Previous reports have shown that Fe‐specific transporters limit the uptake of Cd in yeast (Lee *et al*., 2009; Murata *et al*., 2006, 2008), but this is the first time that a Cd decrease has been observed directly in the seeds of plants exposed to high levels of Cd in the environment. We investigated Cd uptake, translocation and seed loading in *HvYS1* lines with Cd supplied in the soil. The expression of *HvYS1* reduced Cd levels by 2.3‐fold in roots, 5‐fold in leaves and 2.3‐fold in seeds. The decrease in Cd seed concentration is particularly important given the simultaneous 2‐fold increase in Fe levels, because such an approach would simultaneously address the issues of Fe deficiency and Cd toxicity in rice fields with low‐Fe/high‐Cd soils (Clemens *et al*., 2013; Slamet‐Loedin *et al*., 2015). Our results show that plants can take up more Fe in the presence of Cd, and Fe acquisition in the presence of Cd may thus act as a defence mechanism to mitigate Cd‐induced stress (Astolfi *et al*., 2014; Meda *et al*., 2007). Similarly, overexpression of the plastid Fe transporter gene *NtPIC1* in tobacco boosted the Fe/Cd ratio in leaves and improved Cd tolerance (Gong *et al*., 2015), and rice expressing *HvNAS1* and *OsNAS1 + HvNAATb* also accumulated more Fe but less Cd in the seeds compared to wild‐type plants (Masuda *et al*., 2012; Banakar *et al.,* under review). In contrast, rice plants exposed to Fe deficiency in the presence of excess Cd accumulated more Cd in the seeds (Nakanishi *et al*., 2006). The specific uptake, translocation and seed loading of Fe by the *HvYS1* transgenic plants therefore appear to inhibit the uptake, translocation and loading of Cd.

**Figure 6:**
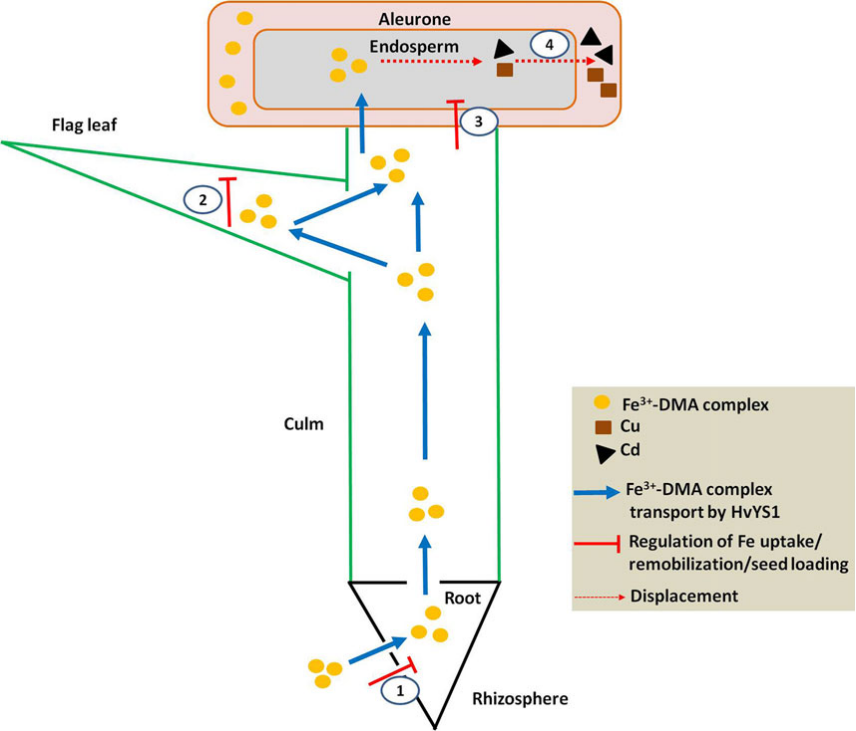
The mechanistic basis of selective Fe transport by HvYS1. Heterologous expression of HvYS1 results in the selective uptake, translocation, remobilization and seed loading of Fe. Endogenous Fe homeostasis limits Fe accumulation in seeds to rather modest levels (i.e. twofold) by modulating the expression of endogenous genes controlling Fe uptake (1), remobilization (2) and seed loading (3), but this is sufficient to displace the toxic heavy metals Cd and Cu from the endosperm (4).

Our findings can be summarized in the mechanistic model presented in Figure 6, which shows that the constitutive expression of *HvYS1* in rice selectively increases the uptake of Fe leading to higher levels of Fe in the roots, followed by selective root‐to‐shoot translocation increasing the Fe concentration in the leaves, promoting the remobilization of Fe from flag leaves and ultimately causing the selective accumulation of Fe in seeds. Iron homeostasis in the roots, leaves and seeds imposes a limit on the concentration of Fe in the seeds (2‐fold when compared with the wild‐type level) through the modulation of endogenous metal transporters, PS synthesis and the Fe storage protein ferritin. The selective mobilization of Fe by HvYS1 has no impact on Zn, Mn and Cu in most tissues, but displaces Cu and Cd from the seeds and Cd from other tissues, providing a strategy for the selective modulation of different metal ions.

In conclusion, we have shown that the heterologous expression of *HvYS1* in rice increases Fe uptake, translocation and seed loading without affecting the uptake, translocation or seed loading of Zn and Mn, without affecting the uptake and translocation of Cu but nevertheless displacing this metal from the endosperm. The concentration of Fe in the seeds of the *HvYS1* transgenic plants is limited to double the normal level, reflecting feedback from the endogenous Fe homeostasis machinery as demonstrated by the modulation of genes controlling endogenous metal transporters and the Fe storage protein ferritin. In contrast to Zn, Mn and Cu, all of which are micronutrients required for the biological activity of certain enzymes and other proteins, Cd is robustly excluded in the transgenic plants during uptake, translocation and seed loading. Our data provide insight into the molecular basis of ion‐selective metal mobilization in plants, which may have evolved to reduce the impact of stress caused by exposure to toxic heavy metals.

## Materials and methods

### Gene cloning and transformation vectors

The *HvYS1* cDNA (GenBank ID AB214183.1) was cloned from the roots of 2‐week‐old barley plants (*Hordeum vulgare* L. cv. Ordalie) growing in vitro on MS medium without Fe (Murashige and Skoog, 1962). Total RNA was extracted using the RNeasy Plant Mini Kit (Qiagen, Hilden, Germany) and 1 mg of total RNA was reverse‐transcribed using the Omniscript RT Kit (Qiagen). The full‐size cDNA (2037 bp) was amplified by PCR using forward primer HvYS1‐BamHI‐FOR (5′‐AGG ATC CAT GGA CAT CGT CGC CCC GGA CCG CA‐3′) and reverse primer HvYS1‐HindIII‐REV (5′‐AAA GCT TTT AGG CAG CAG GTA GAA ACTTCA TG‐3′). The product was transferred to the pGEM^**®**^‐T Easy vector (Promega, Madison, WI) for sequencing and verification. The *HvYS1* cDNA was then subcloned using the BamHI and HindIII sites and inserted into the expression vector pAL76 (Christensen and Quail, 1996), which contains the maize ubiquitin‐1 (*ubi‐1*) promoter and first intron, and an *Agrobacterium tumefaciens nos* transcriptional terminator. The hygromycin phosphotransferase selectable marker gene was controlled by the *CamV35S* promoter and carried a nos terminator for transcriptional termination.

### Rice transformation

Mature rice seed‐derived embryos (*Oryza sativa* L. cv EYI 105) were cultured and excised as previously described (Sudhakar *et al*., 1998; Valdez *et al*., 1998). After 7 days, the embryos were bombarded with gold particles carrying the *HvYS1* transgene and *hpt* selectable marker on separate vectors, with a 3 : 1 molar ratio (Christou *et al*., 1991). The rice embryos were incubated on high‐osmoticum medium (0.2 m mannitol, 0.2 m sorbitol) for 4 h prior to bombardment. Bombarded embryos were selected on MS medium supplemented with 30 mg/L hygromycin, and callus pieces were transferred sequentially to shooting and rooting medium containing hygromycin as above. Regenerated plantlets were transferred to pots containing Traysubstract soil (Klasmann‐Deilmann GmbH, Geeste, Germany) and were grown under flooded conditions in a chamber at 26 ± 2 °C, with a 12‐h photoperiod (900 μmol/m^2^/s photosynthetically active radiation) and 80% relative humidity. Plants were irrigated with a solution of 100 μm Fe provided as Fe (III)‐EDDHA in the form of Sequestrene 138 Fe G‐100 (Syngenta Agro SA, Madrid, Spain).

### RNA blot analysis

Total leaf RNA was isolated using the RNeasy Plant Mini Kit (Qiagen) and 20‐μg aliquots were fractionated on a denaturing 1.2% agarose gel containing formaldehyde before blotting. The membranes were probed with digoxigenin‐labelled partial *HvYS1* cDNA at 50 °C overnight using DIG Easy Hyb (Roche Diagnostics, Mannheim, Germany). After washing and immunological detection with anti‐DIG‐AP (Roche Diagnostics) according to the manufacturer's instructions, CSPD chemiluminescence (Roche Diagnostics) was detected on Kodak BioMax light film (Sigma‐Aldrich, St Louis, MO).

### Cadmium uptake studies

Seeds from three representative transgenic rice lines (1, 2 and 3) were germinated on ½ MS medium supplemented with 50 mg/L hygromycin, and wild‐type seeds were germinated on ½ MS medium without hygromycin. After 7 days, 15 uniform seedlings from wild‐type and transgenic lines were transferred to nutrient solution (Kobayashi *et al*., 2005) containing 10 μm CdCl_2_. The pH of the solution was adjusted to 5.3 with 0.1 m KOH and the plants were maintained as above until seed maturity. Roots, leaves and seeds were harvested from all plants and metal concentrations were quantified by inductively coupled plasma mass spectrometry (ICP‐MS).

### Measurement of metal concentrations by ICP‐MS

Roots and leaves were collected in plastic containers prewashed with 6.5% HNO_3_ to avoid metal contamination. Metals were also removed from the surface of each sample by washing three times in double‐deionized water followed by 100 μm Na_2_EDTA, and EDTA was then removed with two further washes in double‐deionized water. To avoid metal contamination during polishing, dehusked wild‐type and transgenic seeds were polished using a noncontaminating polisher (Kett, Villa Park, CA) and ground using a mortar and pestle prewashed with 6.5% HNO_3_. Roots, leaves and seeds were dried at 70 °C for 2 days and 300‐mg portions were digested with 4.4 m HNO_3,_ 6.5 m H_2_O_2_ and double‐deionized water (3 : 2 : 2) for 20 min at 230 °C using a MarsXpress oven (CEM Corp, Matthews, NC). Metal concentrations were determined in diluted samples by ICP‐MS using an Agilent 7700X instrument (Agilent Technologies, Santa Clara, CA).

### Quantitation of NA and DMA

NA (98% purity) was obtained from Hasegawa Co. Ltd. (Kawasaki, Japan), and DMA (98% purity) was obtained from Toronto Research Chemicals Inc. (Toronto, Canada). Nicotyl‐lysine was synthesized as described by Wada *et al*. (2007). Stock solutions were prepared at concentrations of 1–10 mm and stored in darkness at −80 °C. Working solutions were prepared by diluting the stock solutions with double‐deionized water. Each 5‐μL standard solution was diluted with 5 μL of 50 mm EDTA, 5 μL nicotyl‐lysine and 30 μL of a 1 : 9 ratio mixture of 10 mm ammonium acetate and acetonitrile (pH 7.3), and the mixture was filtered through polyvinylidene fluoride (Durapore^®^ PVDF) 0.45‐μm ultrafree‐MC centrifugal filter devices (Merck KGaA, Darmstadt, Germany) before injection into the HPLC‐ESI‐TOF‐MS system (see below). Fresh root and leaf tissues were extracted as described by Schmidt *et al*. (2011) with some modifications. Samples stored as 200‐mg aliquots at −80 °C prior to extraction were homogenized in 200 μL (roots) or 400 μL (leaves) double‐deionized water containing 36 μL 1 mm nicotyl‐lysine. The homogenate was vortexed for 30 s, sonicated for 5 min and centrifuged at 15 000 ***g*** for 10 min at 4 °C before the supernatant was passed through a 3‐kDa centrifugal filter (cellulose Amicon® Ultra filter units, Merck KGaA). The filtrate was centrifuged as above for 30 min and dried under vacuum. Seeds were ground to a fine powder under liquid N_2_ and extracted three times as described by Wada *et al*. (2007) with some modifications. Aliquots of 50 mg seed powder were extracted in 300 μL double‐deionized water containing 18 μL of 1 mm nicotyl‐lysine. The supernatant was recovered by centrifugation at 15 000 ***g*** for 15 min at 4 °C and stored at −20 °C, and the pellet was extracted twice as above. The three supernatant fractions were pooled and the total extract was passed through the centrifugal filter, centrifuged again and concentrated under vacuum as described above. The dry residues from the leaf/root and seed extracts were dissolved in 20 and 10 μL of type I water, respectively. Then, 5‐μL aliquots of extracts were diluted with 10 μL of 50 mm EDTA, 15 μL type I water and 30 μL of a 1 : 9 ratio mixture of 10 mm ammonium acetate and acetonitrile (pH 7.3), and the mixture was filtered through 0.45‐μm polyvinylidene fluoride (PVDF) ultrafree‐MC centrifugal filter devices (Merck KGaA, Darmstadt, Germany) before analysis.

NA and DMA levels were determined by high‐performance liquid chromatography electrospray ionization time‐of‐flight mass spectrometry (HPLC‐ESI‐TOF‐MS) as described by Xuan *et al*. (2006), with modifications. Details of HPLC conditions are described in SI Materials and Methods, and the details of TOF‐MS operating conditions are listed in Table S2.

### Quantitation of endogenous gene expression

Quantitative real‐time RT‐PCR was carried out to measure steady state mRNA levels in roots, flag leaf and immature seeds, representing the endogenous genes listed in Table S1. Due to its stable expression, actin is a reliable reference gene for qRT‐PCR studies (Cheng *et al*., 2007; Lee *et al*., 2011). Hence, *OsActin1* was used as a reference gene (details of PCR conditions are described in SI Materials and Methods).

## Author contributions

R.B., A.A.F. and P.C. designed the research; R.B. performed the research; R.B and A.A.F. analysed the data; R.B., A.A.F., J.A., T.C and P.C. wrote the manuscript.

## Conflict of interest

Authors declare no conflict of interest.

## Supporting information


**Figure S1** Quantitative real‐time PCR analysis of *OsIRT1, OsYSL15, OsNRAMP5, OsVIT1, OsYSL2, OsYSL16, OsFRDL1, OsYSL18, OsNRAMP1, OsFERRITIN1, OsSAMS1, OsNAS2, OsNAS3, OsNAAT1, OsDMAS1 and OsIDEF1* in roots (left), flag leaf (centre) and seeds (right) at grain filling stage in wild‐type (WT) and T_2_ generation transgenic lines expressing *HvYS1* (Line 1 and Line 2). Each value is the average of three independent experiments. Transcript levels are represented by the ratio between mRNA levels of *OsIRT1, OsYSL15, OsNRAMP5, OsVIT1, OsYSL2, OsYSL16, OsFRDL1, OsYSL18, OsNRAMP1, OsFERRITIN1, OsSAMS1, OsNAS2, OsNAS3, OsNAAT1, OsDMAS1 and OsIDEF1* and those of *OsACTIN1*. Asterisks indicate a statistically significant difference between wild‐type and transgenic plants as determined by Student's *t*‐test (*P* < 0.05; *n* = 3). Gene‐specific primers are listed in Table S1.
**Table S1** Genes and primers used for quantitative real‐time RT‐PCR analysis.
**Table S2** Operating conditions of the time‐of‐flight (TOF) mass spectrometer (MS) used for NA and DMA determinations.
**Data S1** Materials and Methods.
