# Supplementary material for: The expression of heterologous Fe (III) phytosiderophore transporter HvYS1 in rice increases Fe uptake, translocation and seed loading and excludes heavy metals by selective Fe transport
Source: Plant Biotechnol J. 2016 Oct 10;15(4):423–32. doi: 10.1111/pbi.12637 (PMC5362680; doi:10.1111/pbi.12637)
Supplement: Supplementary file 1 — Figure S1 Quantitative real‐time PCR analysis of OsIRT1, OsYSL15, OsNRAMP5, OsVIT1, OsYSL2, OsYSL16, OsFRDL1, OsYSL18, OsNRAMP1, OsFERRITIN1, OsSAMS1, OsNAS2, OsNAS3, OsNAAT1, OsDMAS1 and OsIDEF1 in roots (left), flag leaf (centre) and seeds (right) at grain filling stage in wild‐type (WT) and T2 generation transgenic lines expressing HvYS1 (Line 1 and Line 2). Each value is the average of three independent experiments. Transcript levels are represented by the ratio between mRNA levels of OsIRT1, OsYSL15, OsNRAMP5, OsVIT1, OsYSL2, OsYSL16, OsFRDL1, OsYSL18, OsNRAMP1, OsFERRITIN1, OsSAMS1, OsNAS2, OsNAS3, OsNAAT1, OsDMAS1 and OsIDEF1 and those of OsACTIN1. Asterisks indicate a statistically significant difference between wild‐type and transgenic plants as determined by Student's t‐test (P < 0.05; n = 3). Gene‐specific primers are listed in Table S1. Table S1 Genes and primers used for quantitative real‐time RT‐PCR analysis. Table S2 Operating conditions of the time‐of‐flight (TOF) mass spectrometer (MS) used for NA and DMA determinations. Data S1 Materials and Methods. [file PBI-15-423-s001.doc]

**Table S1:** Genes and primers used for quantitative real-time RT-PCR analysis

| Gene function | Full name of gene | Abbreviated gene name | Gene Bank ID | Amplicon  (bp) | Forward primer | Reverse primer |
| --- | --- | --- | --- | --- | --- | --- |
| Metal transporter | Yellow stripe like 15 Fe (III)-DMA transporter 15 | *OsYSL15* | AB190923.1 | 78 | CTGGTGCTGTTTGCTTGGA | ATGAGCCCAGACGCAACAGC |
|  | Fe regulated transporter 1 | *OsIRT1* | BAB85123.1 | 139 | GCATCATGCAATTCGCTGC | CCTGAACAACCACGCTACAA |
|  | natural resistance-associated macrophage proteins 5 | *OsNRAMP5* | AB690551.1 | 151 | GCTGCCGTTTGCTCTCATCC | CCGATGATGAGCAGACCCAGGA |
| Vacuolar sequestration | Vacuolar iron transporter 1 | *OsVIT1* | NM_001059545.1 | 107 | CCACAGCACAGAACGCCAT | GCTGAGGAATGGACGGTTT |
| Long distance transport | natural resistance-associated macrophage proteins1 | *OsNRAMP1* | DQ431468.1 | 98 | CGGTCGTCTCATCGTCATTGC | ACCTATCTTGTTGCTGCTGCTGC |
|  | Yellow stripe like 2 transporter | *OsYSL2* | AB164646 | 169 | GGTGGAGAGAGTTGTGGGTT | TCATTCCCGCACCAACATA |
|  | Yellow stripe like 16 transporter | *OsYSL16* | AB673449.1 | 157 | GGCTCTACTGGACTGCTTTCG | GCTGCGACAAAGAAGACCG |
|  | Ferric redictase defective citrate extrusion transporter 1 | *OsFRDL1* | NM_001055921.1 | 110 | GCATTCCTTTTGTCGCTGG | GGAGATAGCAGCCACACCA |
|  | Yellow stripe like 18 transporter | *OsYSL18* | NM001051227.1 | 135 | CGGAGTTCGGTCGGATGATG | TTGATGAGGTGAGCCGTCGC |
| Iron storage | *Ferritin1* | *OsFERRITIN1* | AF519570.1 | 130 | GCTTGAATGGAGGAGACTGTG | CTCCATTGCTACTGCGTGCT |
| Transcription factor | Iron deficiency responsive element recognition transcription factor1 | *OsIDEF1* | AK107456 | 90 | GTCTTCAGGCTGGG  GATGT | GGGATTTGTCTGCTGA  TG |
| Phytosiderophore synthesis pathway | S-adenosylmethionine synthase1 | *OsSAMS1* | U82833.1 | 80 | GTCCTCATCTCCACCCAGCA | GAGGGGTTGAGGTGGAAGAT |
|  | Nicotianamine synthase2 | *OsNAS2* | NM_001056429.1 | 134 | GTCCTCATCTCCAC  CAGCA | GAGGGGTTGAGGTGG  AAGAT |
|  | Nicotianamine synthase3 | *OsNAS3* | NM_001067242.1 | 100 | GCACCAGAAGATGG  AGGACA | TGGTGAGGTAGCAAGC  GATG |
|  | Nicotianamine aminotransferase1 | *OsNAAT1* | AB182275.1 | 112 | GATGGCGACTTGG  TTGGGT | GCTCCCTGAATGAAAG  TTGCT |
|  | Deoxymugenic acid synthase1 | *OsDMAS1* | AB269906 | 149 | TCCAAGGGCAAGACCGTAG | ATCCTCTGCCTCTCCTC  CTC |
| Reference gene | *Actin1* | *OsACTIN1* | AB047313.1 | 120 | TCA TGT CCC TCA CAA TTT CC | GAC TCT GGT GAT GGT GTC AGC |

**Table S2:** Operating conditions of the time-of-flight (TOF) mass spectrometer (MS) used for NA and DMA determinations

| Source | Electrospray |
| --- | --- |
| Polarity | Negative |
| Endplate voltage | -0.5 kV |
| Spray tip voltage | 3.0 kV |
| Orifice voltage | 107 V |
| Nebulizer gas | N_2_ |
| Nebulizer gas pressure | 2.0 bar |
| Drying gas | N_2_ |
| Drying gas (N_2_) flow rate | 8.5 L min^-1^ |
| Drying gas temperature | 180 ºC |

**
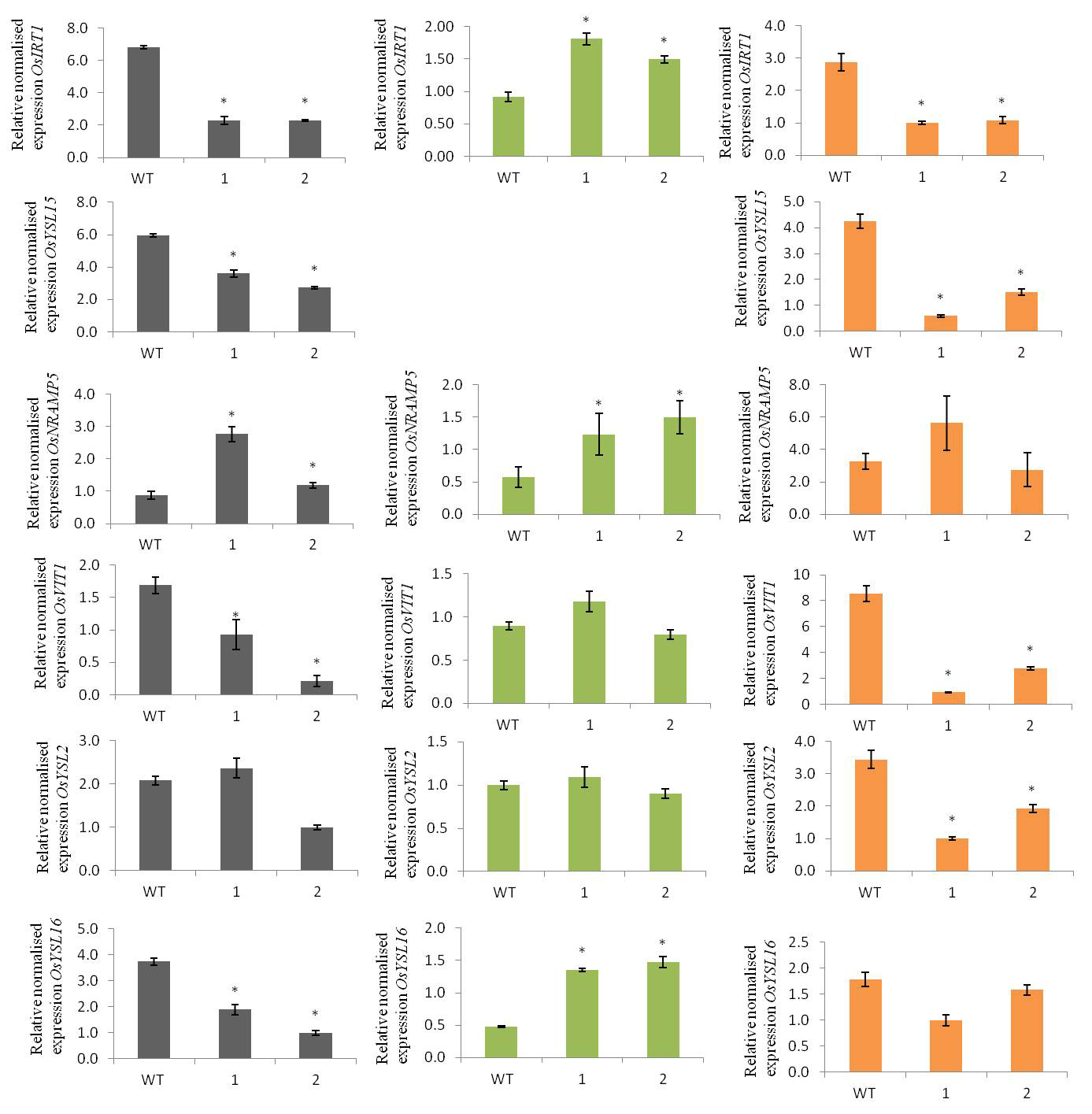
**

**
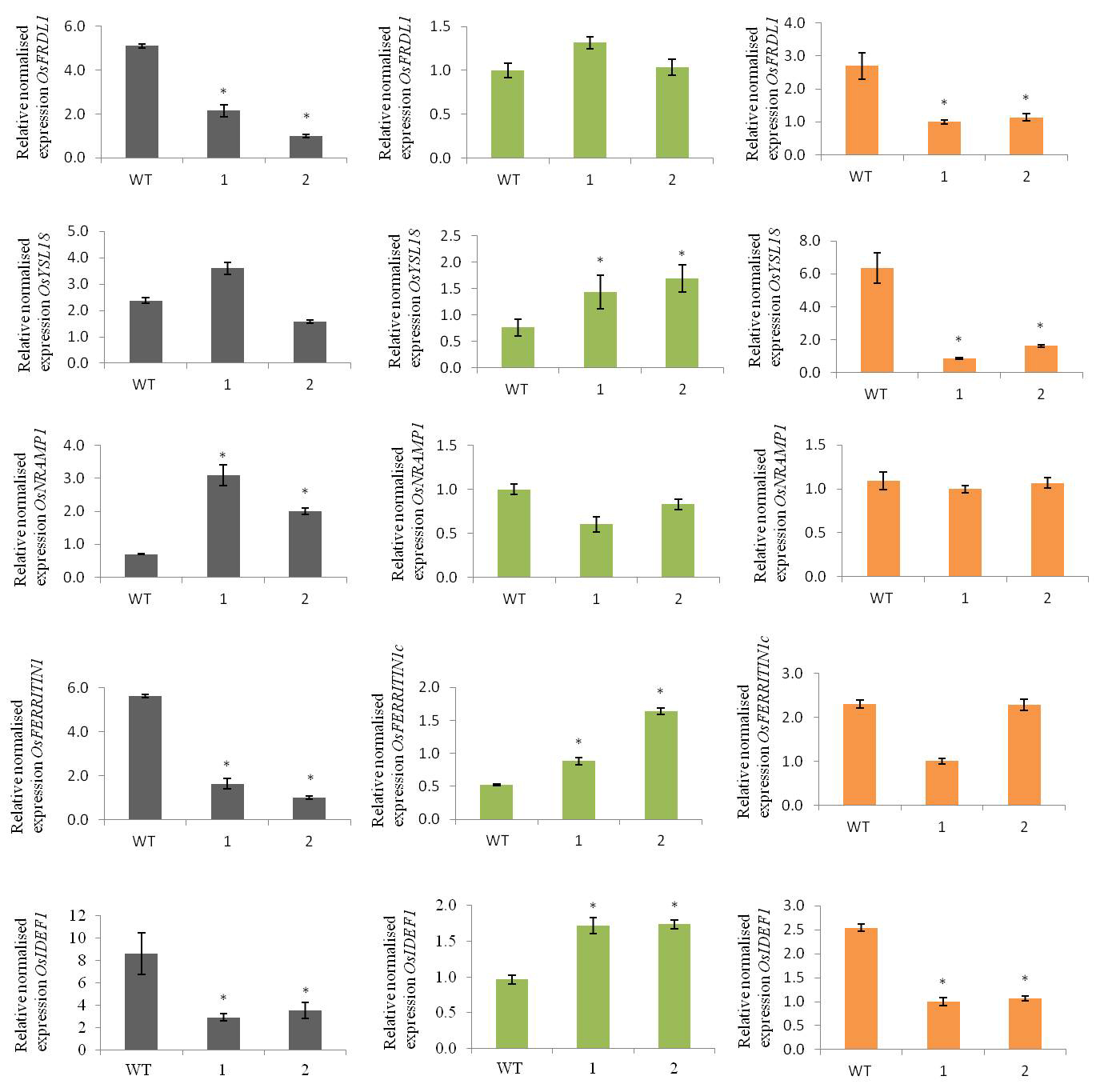
**

**
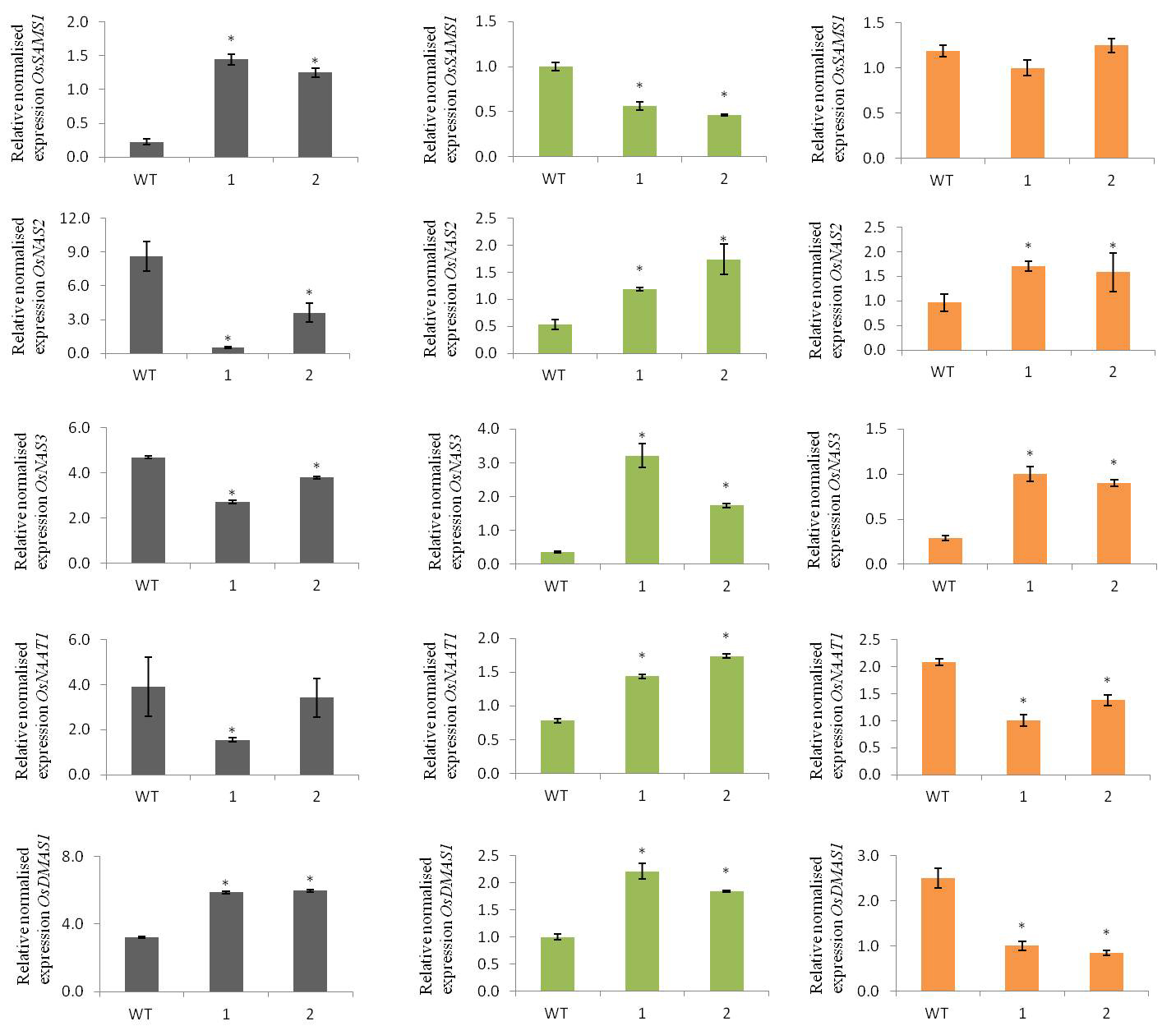
**

**Figure S1:** quantitative Real-time PCR analysis of *OsIRT1, OsYSL15, OsNRAMP5, OsVIT1, OsYSL2, OsYSL16, OsFRDL1, OsYSL18, OsNRAMP1, OsFERRITIN1,OsSAMS1, OsNAS2, OsNAS3, OsNAAT1, OsDMAS1, OsIDEF1* in roots (left), flag leaf (centre) and seeds (right) at grain filling stage in wild type (WT) and T_2_ generation transgenic lines expressing *HvYS1* (Line 1 and Line 2). Each value is the average of three independent experiments. Transcript levels are represented by the ratio between mRNA levels of *OsIRT1, OsYSL15, OsNRAMP5, OsVIT1, OsYSL2, OsYSL16, OsFRDL1, OsYSL18, OsNRAMP1, OsFERRITIN1, OsSAMS1, OsNAS2, OsNAS3, OsNAAT1, OsDMAS1, OsIDEF1* and those of *OsACTIN1*. Asterisks indicate a statistically significant difference between wild type and transgenic plants as determined by Student’s t test (p <0.05; n=3). Gene-specific primers are listed in Supplementary Table S1.

**Supplementary Materials and Methods**

*Quantitation of NA and DMA*

The samples were fractionated using an Alliance 2795 HPLC system (Waters, Milford, MA, USA) and µLC column (SeQuant ZIC®-HILIC, 15 cm x 1 mm internal diameter, 5 µm, 200 Å, Merck KGaA), with a mobile phase consisting of solvent A (9:1 10 mM ammonium acetate:acetonitrile, pH 7.3) and solvent B (8:2 30 mM ammonium acetate:acetonitrile, pH 7.3) at a flow rate of 0.15 mL min^-1^. The gradient program started at 100% solvent A for 3 min, and then decreased linearly to 30% solvent A over the next 7 min, then remained for 7 min at 30% solvent A, and then returned to the initial conditions over the next 8 min. The column was then allowed to stabilize for 10 min at the initial conditions before proceeding to the next injection. The total HPLC run time was 35 min, the injection volume was 10 µL and the auto sampler and column temperatures were 6ºC and 30ºC, respectively. The HPLC was coupled to the MicrOTOF mass spectrometer (Bruker Daltonics, Bremen, Germany) equipped with an ESI source. The operating conditions were optimized by the direct injection of 100 µM solutions of NA and DMA standards at a flow rate of 180 µL h^-1^. Mass spectra were acquired in negative ion mode (**Table S2**) over the 150­–700 *m/z* range. The mass axis was calibrated externally and internally using Li–formate adducts (10 mM LiOH, 0.2% (v/v) formic acid and 50% (v/v) 2-propanol). Bruker Daltonik software packages micrOTOF Control v2.2, HyStar v3.2 and Data Analysis v4.0 were used to control the MS, HPLC interface and for data processing, respectively. NA and DMA concentrations were quantified by external calibration, using nicotyl-lysine as internal standard.

*Quantitation of endogenous gene expression*

The samples were amplified using a BioRad CFX96^TM^ system and 25-µL mixtures containing 10 ng of cDNA, 1x iQSYBR Green supermix (BioRad, Hercules, CA, USA) and 0.2 μM forward and reverse primers (**Table S1**). Relative expression levels were calculated on the basis of serial dilutions of cDNA (125–0.2 ng) and used to generate standard curves for each target gene*.* Each experiment was carried out in triplicate using Hard-Shell® 96 Well-Semi-Skirted PCR plates (BioRad). Cycling conditions consisted of a single incubation step at 95°C for 5 min followed by 39 cycles of 95°C for 10 s, 59.4°C for 35 s and 72°C for 15 s. Speciﬁcity was conﬁrmed by product melt curve analysis over the temperature range 50–90°C with ﬂuorescence acquired after every 0.5°C increase. The ﬂuorescence threshold value and gene expression data were calculated using BioRad CFX96^TM^ software. Values represent the mean of three RT-PCR replicates ± SE. Ampliﬁcation efficiencies were compared by plotting the ΔCt values of different primer combinations in serial dilutions against the log of the starting template concentrations using the CFX96^TM^ software.
